# The Relationship between Health-Related Fitness and Quality of Life in Nonalcoholic Fatty Liver Disease

**DOI:** 10.3390/ijerph192114215

**Published:** 2022-10-31

**Authors:** Lina Wang, Jing Zhang, Yali Liu, Huixuan Zhou, Wenjing Yan, Hong Ren

**Affiliations:** 1 School of Sport Science, Beijing Sport University, Beijing 100084, China; 2 The Third Unit, Department of Hepatology, Beijing Youan Hospital, Capital Medical University, Beijing 100069, China; 3 School of Physical Education, Shanxi Normal University, Taiyuan 030000, China

**Keywords:** nonalcoholic fatty liver disease, fitness, quality of life, cardiorespiratory fitness, body composition

## Abstract

Background: It is well known that patients with nonalcoholic fatty liver disease (NAFLD) suffer from impaired quality of life (QoL) and decreased health-related fitness. Studies on the relationship between them have been scarce. Methods: A cross-sectional survey was performed in 104 NAFLD patients. Liver fat content and fibrosis were assessed using transient elastography. Health-related fitness was measured by fitness test. VO_2_max was determined by YMCA submaximal cycle ergometer test. Body composition was tested by bioimpedance analysis. QoL was evaluated using the 36-item Short Form Health Survey Questionnaire (SF-36). Results: Most patients had severe liver steatosis without significant fibrosis. Most of them exhibited poor health-related fitness. Multiple linear regression analyses demonstrated that body compositions (waist circumference, hip circumference, percent body fat, percent skeletal muscle, visceral fat area) dependently contributed to QoL (health transition, role limitation due to physical problem, general health, physical functioning and vitality). VO_2_max was positively related with physical functioning. Conclusion: For NAFLD patients, decreased health-related fitness was associated with impaired QoL both in the physical and mental dimension. Our results indicate that visceral fat together with muscle mass and VO_2_max could serve as individual exercise intervention targets to improve QoL.

## 1. Introduction

Non-alcoholic fatty liver disease (NAFLD) is a highly prevalent liver disease affecting about 25% of the adult population worldwide [1]. In addition to the serious outcomes, such as end-stage liver disease and promoting incidence of metabolic trait, NAFLD also decreases the quality of life (QoL) [2]. In our outpatient group, a large proportion of NAFLD patients sought medical advice due to symptoms associated with low QoL.

QoL is a broad ranging concept affected in a complex way by a person’s physical health, psychological state, level of independence, social relationships and their relationship to salient features of their environment [3]. Previous studies have shown that patients with NAFLD suffered a lot from the impaired QoL [4,5,6]. The prominent manifestation is fatigue, which is not paralleled with liver function [7]. The determinants of QoL are complex, including disease and treatment, health care provision or psychological variables [8]. It is well accepted that health-related fitness is closely associated with QoL in the general population [9,10].

Health-related fitness usually includes cardiorespiratory fitness (CRF), muscular strength and endurance, body composition and flexibility. It has been reported that patients with NAFLD present reduced CRF and muscular strength [11]. However, whether reduced health-related physical fitness is also a cause of impaired QoL has not been fully studied.

Therefore, we carried out the cross-sectional study to investigate the association between health-related fitness and QoL in NAFLD patients. The results may help to discover the additional cause of low QoL and facilitate development and implementation of specific exercise intervention to improve QoL. 

## 2. Materials and Methods

### 2.1. Study Design

This was a single-center cross-sectional study from October 2021 to September 2022. The guidelines on ‘Strengthening the Reporting of Observational Studies in Epidemiology (STROBE)’ were chosen to guide this study. The study was in accordance with the ethical guidelines of the 1975 Declaration of Helsinki and approved by the Ethical Committee of Beijing Youan Hospital (approval number: 2018-095). All patients provided written informed consent form to have their data used (anonymously) for research purposes.

### 2.2. Participants

**Figure 1:**
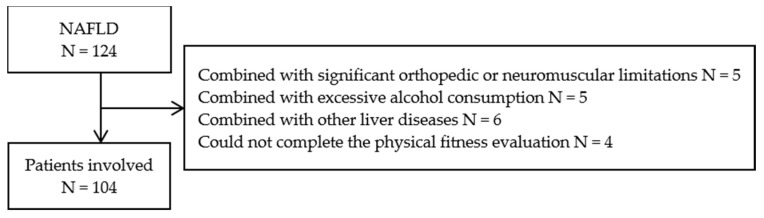
Flow chart of patients’ selection.

NAFLD patients came from the outpatient clinic of Beijing Youan Hospital. Prior to enrolment, eligible patients had a medical screen to exclude uncontrolled cardiopulmonary disease or other contraindications to exercise testing as outlined in the American College of Sports Medicine guidelines [12]. The inclusion criteria were: (1) age ≥18 years; (2) fatty liver diagnosed by B-type ultrasound or liver biopsy. The exclusion criteria were: (1) contraindications to exercise testing; (2) significant orthopedic or neuromuscular limitations; (3) unwillingness to participate; (4) alcohol consumption ≥30 g/ day (males) or ≥20 g/day (females); (5) coexisting with other liver diseases, such as hepatitis B and C, autoimmune liver diseases, secondary fatty liver due to specific drugs, etc.; (6) could not complete the physical fitness evaluation. Finally, 124 cases were screened, and 104 patients were included (Figure 1).

### 2.3. Data Collection

#### 2.3.1. NAFLD Definition and Classification

The presence of fatty liver was assessed by B-type ultrasound. Partial patients were confirmed by liver biopsy. Liver fat content and fibrosis were measured by Fibroscan 502 touch device (Echosens, Paris, France). As to the value of controlled attenuated parameter (CAP), liver steatosis was classified into 3 grades: mild liver steatosis: 238–258 db/m, moderate liver steatosis: 259–292 db/m, severe liver steatosis: >292 db/m. Liver stiffness measurement (LSM) was used to assess the severity of liver fibrosis.

#### 2.3.2. Assessment of Quality of Life

QoL was assessed using the 36-item Short Form Health Survey Questionnaire (SF-36), its reliability and validity have been verified on Chinese in mainland China [13]. SF-36 is a multipurpose and short-form health survey, which is commonly used to evaluate patients’ QoL in clinical practice, including NAFLD patients [14,15,16]. A total of eight domains were evaluated in this questionnaire, including physical functioning (PF), role limitation due to physical problems (RP), bodily pain (BP), general health (GH), vitality (VT), social functioning (SF), role limitation due to emotional problem (RE) and mental health (MH). Additionally, the eight health domains can be used to provide a physical component summary score (PCS) and a mental component summary score (MCS). In addition to these eight domains, patients’ health transition (HT) will also be investigated. Each domain is scored from 0 to 100, so that the lowest score represents low QoL and vice versa [17].

#### 2.3.3. Assessment of Health-Related Fitness

Muscular strength was tested using grip strength (GS). GS was detected twice by hand grip dynamometer (EH101, CAMRY, Guangdong, China) with the dominant hand. The higher value was recorded as GS [18]. Relative grip strength (RGS) is the ratio of grip strength to BMI [19]. BMI was calculated using the Quetlet’s formula: BMI = Weight (kg)/Height (m)^2^. Obesity was defined as BMI ≥ 25 kg/m^2^ [20]. The results were evaluated according to the National Physical Fitness Evaluation Standard (2003) [18]. 

Cardiorespiratory fitness test was carried out using the YMCA power car scheme (YMCA Submaximal Cycle Ergometer Test) [21]. The stable heart rates of study participants were controlled between 110 beats per minute (bpm) and 70% of the heart rate reserve (85% of the age-predicted heart rate) in the continuous tests, and the heart rates at the 45–60 s of the 2nd and 3rd minute were recorded. If the heart rate changed more than 5 bpm, the movement was extended for 1 min at this power. The heart rate and power of 2 points in continuous stages with stable heart rates of 110 bpm or more were selected as a straight line. The extended line of the straight line was used to determine the predicted maximum power corresponding to the age-predicted maximum heart rate, and then the VO_2_max was calculated according to the standard formula. The results were evaluated according to Leonard A. Kaminsky’s study [22].

Flexibility was assessed using sitting forward flexion (SFF) by sitting on a flexibility measuring instrument (TQQ-IIA, XDHT, Beijing, China) with their heels positioned at the edge of the device. Patients then bent forward at the waist with their hands outstretched in front of them to push the measuring instrument as far as possible past their feet. The better distance of two tests was recorded. The results were evaluated according to the National Physical Fitness Evaluation Standard (2003) [18].

Percent body fat (PBF), skeletal muscle mass and visceral fat area (VFA) were estimated by a bioimpedance analysis (InBody720, InBody, Seoul, Korea). Percent skeletal muscle (PSM) is the ratio of skeletal muscle mass to the total weight. 

Waist circumference (WC) is one of the indicators of central obesity [23], and it was measured at the midpoint between the lower rib cage and iliac crest. The hip circumference (HC) was measured at the level of maximum extension of the buttocks while the participant stood upright with the feet held together. WC and HC were measured using a non-stretchable fiberglass tape [24]. The waist-to-hip ratio (WHR) was calculated as waist circumference divided by hip circumference. Central obesity was defined as waist circumference (≥90 cm for men and ≥80 cm for women) [25].

#### 2.3.4. Assessments and Definitions of Other Variables

Demographic data, past medical history and laboratory data including liver function, fasting blood sugar (FBS) and blood lipid profile were collected. T2DM was diagnosed when: FBS ≥ 7.0 mmol/L, HbA1c ≥ 6.5% or OGTT 2 h blood sugar ≥ 11.1 mmol/L, or taking hypoglycemic drugs [26]. Dyslipidemia was diagnosed when elevated blood lipids met at least one of the following: TC ≥ 6.22 mmol/L, TG ≥ 2.26 mmol/L, LDL-C ≥ 4.14 mmol/L or HDL-C < 1.04 mmol/L or under antihyperlipidemic treatment [27]. Hyperuricemia was defined as serum uric acid above 420.0 µmol/L in males and 360.0 µmol/L in females or use of uric acid lowering drugs [28]. Hypertension was defined as an average systolic blood pressure ≥140 mmHg or average diastolic blood pressure ≥90 mmHg or as the use of antihypertension medication [29].

### 2.4. Statistical Analysis

Data analyses were performed using the Statistical Package for the Social Sciences for Windows (v 26.0, SPSS Inc., Chicago, IL, USA). Normal distribution continuous data were presented as the mean ± standard deviation. Differences in variables between genders and grades of liver steatosis were calculated using independent-sample t test. Percentages were used to summarize categorical variables, and data were compared by chi-squared test. Trends and associations between health-related fitness and QoL were evaluated using Pearson’s analysis of correlation. To identify potential predictive factors for QoL, multiple linear regression analyses were performed using a stepwise procedure. All dimensions of QoL were selected and represented as dependent variables. Statistically significant variables of health-related fitness after Pearson correlation were selected as independent variables and were adjusted for sex, age, comorbidity and degree of liver steatosis. A *p*-value of <0.05 was considered statistically significant. 

## 3. Results

### 3.1. Characteristics of Patients

**Figure 2:**
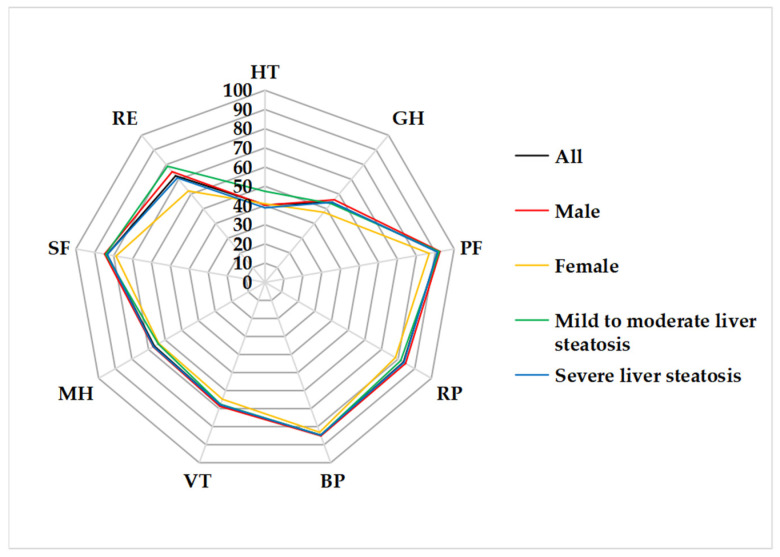
Radar chart of QoL scores in different dimensions with NAFLD patients.

A total of 124 NAFLD patients were screened, and 104 cases were enrolled in this study. The mean age was 37.0 ± 9.7 years. Male patients accounted for 78.8%. Mean BMI was 28.6 ± 3.8 kg/m^2^. The proportion of severe hepatic steatosis in men and women were 81.7% and 81.8%, respectively (Table 1). Compared with other dimensions of QoL, HT and GT scores are relatively low (Figure 2). 

### 3.2. Characteristics of Health-Related Fitness 

VO_2_max levels were lower than average in 91.5% of males and 80.0% of female NAFLD patients. There were 48.8% of male patients and 50% of female patients with GS lower than grade 3 (medium level). Meanwhile, there were 86.6% of male patients and 59.1% of female patients with SFF lower than grade 3 (medium level). No patient achieved grade 5 of SFF (Table 2).

The dimensions of QoL, including GH, HT, PF, RP, PCS and VT were found to be correlated with indicators of health-related fitness. The correlations were listed in Table 3.

As listed in Table 4, WC, PBF, PSM and VO_2_max, HC, PSM and VFA contributed significantly to HT, GH, PF, RP, PCS and VT with an adjusted beta of −0.278, −0.337, 0.321 and 0.211, 0.230, 0.215 and −0.200, respectively.

## 4. Discussion

NAFLD is a highly prevalent liver disease which affects about one quarter of adults worldwide. In addition to the risk of progression to end-stage liver disease, NAFLD can also impact life quality in many ways in the early stage. It has been reported that demographics, clinical parameters and severity of disease were all related to QoL [8]. Few studies have explored the influence of health-related fitness on QoL of NAFLD patients. From our research, we discovered that the majority of patients exhibited low health-related fitness and QoL. PSM, VO_2_max and HC were positively associated with different physical-related dimensions of QoL; meanwhile, PBF, WC and VFA exhibited negative correlation. VFA was the only indicator that was associated with the mental-related dimension of QoL. These findings deepened our knowledge of the importance of health-related fitness to NAFLD patients and revealed the potential link of exercise intervention with QoL improvement through enhancing health-related fitness. 

The study was performed in the outpatient clinic of a hepatology hospital. Most of NAFLD patients came to the clinic due to occurrence or progression of fatty liver disease, elevation of liver enzymes or symptoms such as fatigue, dull pain of right upper abdomen, etc. After medical assessment by a hepatologist, patients were transferred to an exercise physiologist and acquired exercise intervention after physical fitness assessment. Patients who met the inclusion and exclusion criteria were included in the study. In our cohort, male patients accounted for 78.8% and were much younger than female patients. Obese patients accounted for 84.6%. Most patients had dyslipidemia, while the proportion of T2DM, hypertension and hyperuricemia were relatively low. Most patients had elevated ALT/AST/γ-GT and severe liver steatosis without obvious fibrosis. The manifestations indicated that most of the patients were in the early stage of NAFLD.

In the study, three indicators of health-related fitness were analyzed in detail. VO_2_max is the gold standard for assessing CRF, which is viewed as the fifth vital sign due to the close relationship with the incidence of cardiovascular disease, all-cause mortality, and mortality rates attributable to various cancers [30]. The mean level of VO_2_max was 31.8 ± 5.9 mL/kg/min, and only three cases (3.0%) were above the average level according to the international standard [22]. The fact of low VO_2_max in NALFD was also observed in previous studies. In the study by Li Hao et al., the average VO_2_max was 31.19 ± 4.51 mL/kg/min in 224 male Chinese NAFLD patients, in which VO_2_max was also determined by YMCA test. In an American study, VO_2_max was 26.8 ± 7.4 mL/kg/min which included 37 patients in which VO_2_max was determined by Bruce test [31]. In a UK study, the VO_2_ peak was determined by Cortex metalyser 3B instrument. The mean VO_2_max was 34.4 ± 7.3 mL/kg/min lean body mass in male and 29.0 ± 7.6 mL/kg/min lean body mass in female [32]. In those studies, VO_2_max was significantly lower in NAFLD than in healthy control. Furthermore, we compared VO_2_max between the two groups of patients with mild to moderate and severe liver steatosis, VO_2_max was not influenced by severity of steatosis. The result was similar to the American study. In summary, we deduced that NAFLD exhibited worse CRF but not related with the severity of liver steatosis. The mechanism of CRF reduction in NAFLD was not clear. Afolabi’s study indicated that in NAFLD patients, hepatic mitochondrial dysfunction was related to CRF reduction [30].

GS is one of the indicators of muscle mass and strength. In our study, GS lower than grade 3 (medium level) was seen in 48.8% of male and 50% of female NAFLD patients, indicating that muscle mass and strength reduction was very common in NAFLD patients although their age is relatively young. A Korean study also discovered the relationship between GS and QoL in general population [33]. The association between liver and muscle has been fully studied. Skeletal muscle has been regarded as an important endocrine organ for postprandial glucose utilization and secretion of myokines mediating crosstalk between muscle, liver, adipose tissue, and other organs [34]. Sarcopenia has been verified as one of the risk factors of NAFLD development [35]. In a prospective cohort, low GS and muscle mass contributed to development of severe NAFLD [36]. In their study, severe NAFLD was defined as hospitalization or death due to NAFLD. In our study, patients in both groups were young and all at early stage of NAFLD without fibrosis. It’s reasonable that no difference was found between the mild to moderate and severe liver steatosis group.

Flexibility refers to the intrinsic properties of body tissues that determine maximal joint range of motion without causing injury. Studies have found the relationship between cardiometabolic health and SFF [37]. For many years, flexibility has been classified as a major component of physical fitness by many countries [38]. Flexibility determination is also a basis of exercise intervention. In our study, flexibility was evaluated by sitting forward flexion. There were 86.6% of male and 59.1% of female with SFF lower than grade 3. No patient achieved grade 5 of SFF. The results indicated that stretching exercise should also be additional addressed.

The general concept of quality of life was considered a useful adjunct to traditional concepts of health and functional status [39]. Given the accumulating evidence, NAFLD patients experience an impaired QoL, which is significantly lower than the general population [40] and hepatitis C patients [41]. Lower QoL of NAFLD has been verified to be related with elder age, female, low income and educational level, obesity, T2DM, fibrosis and cirrhosis, but not affected by liver enzymes or serum albumin [8]. In our population, the dimensions were also not affected by steatosis severity. The five dimensions, including HT, GH, PF, RP and VT were positively related with PSM, VO_2_max and HC, while negatively related with WC, PBF and VFA in a dependent way. The results were consistent to a study in an American cohort [40]. They discovered a positive correlation of lean body mass, dry lean mass and skeletal muscle with PF, role limitations due to physical health (equal to RP in the paper), emotional well-being (equal to MH in the paper), emotional problems (equal to RE in the paper) and SF scales. Meanwhile, BMI and body fat mass was proven to be negatively correlated with PF, RP, energy/fatigue (equal to VT in the paper), pain (equal to BP in the paper) and GH scales [40]. In summary of both their research and ours, lower QoL of NAFLD was mainly influenced by increase of fatty issue and decrease of muscle. Interestingly, we found that VFA was an independent factor of VT. As previously reported, lower levels of chronic system inflammation in NAFLD and obesity are the cause of the reduction of VT [42]. The results indicated that for the patients with low VT, intervention aiming to decrease VFA should be emphasized. In addition, we also found that as a marker of aerobic ability, VO_2_max was an independent factor of PF together with PSM. Altogether, exercise focusing on decreasing visceral fat and increasing VO_2_max and muscle mass will benefit both the physical and mental dimensions of QoL.

There were also several limitations of our study. First, this is a single center study with small sample size. Second, this study did not include sociodemographic information, such as educational background, smoking habit, physical activity or medical status. Third, the subjects were all in early stage of NAFLD. Hence, there was no chance to fully analyze the relationship between QoL and NAFLD severity. Because of the limitations, our results need to be further verified in the future.

## 5. Conclusions

In summary, in this study, we show that (1) NAFLD patients’ health-related fitness is poor. (2) Health-related fitness influences quality of life directly, body composition and cardiorespiratory fitness are major risk factors for reduced quality of life. For NAFLD patients with reduced quality of life, exercise interventions should be targeted at reducing visceral fat and increasing muscle mass and VO_2_max.

## Figures and Tables

**Table 1:** Characteristics of patients with NAFLD.

Variables	All (N = 104)	Gender		Degree of Liver Steatosis	
		Male (N = 82)	Female (N = 22)	Mild to Moderate (N = 19)	Severe (N = 85)
Demographic data					
Age (years)	37.0 ± 9.7	34.5 ± 7.4	46.4 ± 11.5 **	38.0 ± 11.2	36.8 ± 9.4
Height (cm)	173.4 ± 8.3	176.5 ± 6.2	162.1 ± 4.5 **	172.7 ± 9.2	173.6 ± 8.2
Weight (kg)	86.1 ± 15.8	90.4 ± 13.9	70.3 ± 11.9 **	84.8 ± 16.2	86.4 ± 15.8
BMI (kg/m2)	28.6 ± 3.8	29.1 ± 3.6	26.7 ± 3.9 **	28.2 ± 3.7	28.6 ± 3.8
Obesity, n (%)	88 (84.6%)	74 (90.2%)	14 (63.6%) **	18 (94.7%)	70 (82.4%)
Central obesity, n (%)	99 (95.2%)	79 (96.3%)	20 (90.9%)	18 (94.7%)	81 (95.3%)
Comorbidities					
T2DM, n (%)	9 (8.7%)	7 (8.5%)	2 (9.1%)	1 (5.30%)	8 (9.40%)
Dyslipidemia, n (%)	78 (75.0%)	62 (75.6%)	16 (72.7%)	14 (73.70%)	64 (75.30%)
Hypertension, n (%)	7 (6.7%)	4 (4.90%)	3 (13.6%)	3 (15.80%)	4 (4.70%)
Hyperuricemia, n (%)	24 (23.1%)	23 (28.0%)	1 (4.5%) **	3 (15.80%)	21 (24.70%)
Laboratory data					
ALT (U/L)	74.5 ± 54.5	83.4 ± 56.4	41.5 ± 28.9 **	54.3 ± 40.4	79.1 ± 56.3
AST (U/L)	43.0 ± 23.8	46.1 ± 25.0	31.4 ± 13.7 **	40.2 ± 26.7	43.6 ± 23.2
γ-GT (U/L)	64.2 ± 65.0	64.1 ± 69.8	64.3 ± 43.7	49.7 ± 42.2	67.4 ± 68.8
ALP (U/L)	81.8 ± 20.4	82.3 ± 20.1	79.7 ± 22.0	79.3 ± 17.4	82.3 ± 21.1
FBS (mmol/L)	5.4 ± 0.7	5.4 ± 0.7	5.5 ± 0.6	5.3 ± 0.4	5.5 ± 0.7
TG (mmol/L)	2.2 ± 2.4	2.3 ± 2.7	1.6 ± 0.6	1.6 ± 0.6	2.3 ± 2.6
TC (mmol/L)	5.3 ± 1.1	5.3 ± 1.2	5.4 ± 0.9	5.3 ± 0.8	5.3 ± 1.2
LDL-C (mmol/L)	1.1 ± 0.3	1.0 ± 0.2	1.3 ± 0.3	1.1 ± 0.2	1.1 ± 0.3
HDL-C (mmol/L)	3.4 ± 2.1	3.4 ± 2.3	3.3 ± 0.9 **	3.2 ± 0.8	3.4 ± 2.3
Uric acid (μmol/L)	425.8 ± 88.5	449.8 ± 81.4	336.4 ± 47.6 **	431.7 ± 87.2	424.4 ± 89.3
CAP (dB/m)	329.8 ± 37.4	331.5 ± 36.9	323.7 ± 39.4	271.3 ± 16.4	342.9 ± 26.5 ^##^
LSM (kPa)	6.4 ± 2.3	6.5 ± 2.4	6.0 ± 1.7	6.3 ± 2.4	6.4 ± 2.3
Health-related fitness					
PBF (%)	31.9 ± 5.7	30.4 ± 4.9	37.5 ± 5.0 **	31.3 ± 4.6	32.0 ± 5.9
VFA (cm^2^)	124.6 ± 39.0	122.5 ± 38.9	132.2 ± 39.5	116.9 ± 27.3	126.3 ± 41.1
PSM (%)	38.2 ± 3.6	39.3 ± 2.9	34.0 ± 2.8 **	38.4 ± 3.1	38.1 ± 3.7
WC (cm)	99.4 ± 10.7	101.5 ± 9.5	91.2 ± 11.3 **	97.7 ± 11.2	99.7 ± 10.7
HC (cm)	105.1 ± 7.1	106.2 ± 6.6	101.2 ± 7.7 **	104.6 ± 7.2	105.2 ± 7.2
WHR	0.9 ± 0.1	1.0 ± 0.0	0.9 ± 0.1 **	0.9 ± 0.1	0.9 ± 0.1
VO_2_max (mL/kg/min)	31.8 ± 5.9	32.1 ± 6.1	30.7 ± 5.1	33.3 ± 7.8	31.4 ± 5.4
RGS	1.2 ± 0.5	1.3 ± 0.5	0.8 ± 0.3 **	1.3 ± 0.4	1.2 ± 0.5
GS (kg)	40.6 ± 10.1	44.3 ± 7.5	26.8 ± 5.4 **	39.6 ± 11.1	40.8 ± 9.9
SFF (cm)	-2.9 ± 8.7	-3.7 ± 8.5	0.0 ± 9.0	0.5 ± 8.4	−3.7 ± 8.7
Quality of life					
HT	40.4 ± 27.3	40.2 ± 25.4	40.9 ± 34.1	47.4 ± 24.9	38.8 ± 27.7
PCS	78.4 ± 12.8	79.5 ± 12.8	74.0 ± 12.2	78.0 ± 11.2	78.4 ± 13.2
GH	54.3 ± 24.6	56.1 ± 26.3	47.6 ± 14.9	53.5 ± 19.8	54.4 ± 25.6
PF	91.3 ± 7.8	92.5 ± 7.2	86.8 ± 8.2 **	92.1 ± 5.8	91.1 ± 8.1
RP	83.2 ± 29.4	84.5 ± 28.5	78.4 ± 33.0	81.6 ± 31.0	83.5 ± 29.3
BP	84.7 ± 16.4	85.1 ± 17.2	83.1 ± 13.4	84.7 ± 16.4	85.1 ± 17.2
MCS	72.6 ± 16.6	74.0 ± 15.7	67.4 ± 19.1	73.8 ± 16.7	72.3 ± 16.7
MH	66.5 ± 15.7	67.2 ± 14.7	63.8 ± 18.9	64.2 ± 20.1	67.0 ± 14.6
VT	67.9 ± 15.9	68.7 ± 15.3	64.8 ± 17.9	67.6 ± 21.4	67.9 ± 14.5
SF	83.5 ± 15.6	84.8 ± 15.4	78.8 ± 16.0	84.2 ± 15.4	83.4 ± 15.8
RE	72.4 ± 36.7	75.2 ± 35.1	62.1 ± 41.5	78.9 ± 33.7	71.0 ± 37.4

** *p* < 0.01, comparison between female and male group. ^##^ *p* < 0.01, comparison between mild to moderate group and severe liver steatosis group.

**Table 2:** Grades of health-related fitness in NAFLD patients (N = 104).

Variables	Proportion (%)					χ2	P
VO_2_max							
Gender	Poor	Fair	Average	Good	Excellent	6.456	0.168
Male, n (%)	57 (69.5%)	18 (22.0%)	5 (6.1%)	0 (0.0%)	2 (2.4%)		
Female, n (%)	12 (60.0%)	4 (20.0%)	3 (15.0%)	1 (5.0%)	0 (0.0%)		
All, n (%)	69 (67.6%)	22 (21.6%)	8 (7.8%)	1 (1.0%)	2 (2.0%)		
Grip strength							
Gender	Grade 1	Grade 2	Grade 3	Grade 4	Grade 5	2.455	0.783
Male, n (%)	16 (19.5%)	22 (26.8%)	28 (34.1%)	9 (11.0%)	5 (6.1%)		
Female, n (%)	3 (13.6%)	8 (36.4%)	6 (27.3%)	4 (18.2%)	1 (4.5%)		
All, n (%)	19 (18.3%)	30 (28.8%)	34 (32.7%)	13 (12.5%)	6 (5.8%)		
Sitting forward flexion							
Gender	Grade 1	Grade 2	Grade 3	Grade 4	Grade 5	13.015	0.011
Male, n (%)	22 (26.8%)	23 (28.0%)	6 (7.3%)	5 (6.1%)	0 (0.0%)		
Female, n (%)	4 (18.2%)	3 (13.6%)	8 (36.4%)	1 (4.5%)	0 (0.0%)		
All, n (%)	26 (25.0%)	26 (25.0%)	14 (13.5%)	6 (5.8%)	0 (0.0%)		

P for comparison between female and male group.

**Table 3:** Correlation between health-related fitness and QoL (N = 104).

Variables	HT	PCS	GH	PF	RP	BP	MCS	MH	VT	SF	RE
BMI	−0.193 *	0.000	−0.187	−0.102	0.211 *	−0.051	0.009	0.035	−0.054	−0.048	0.045
PBF	−0.199 *	−0.197 *	−0.337 **	−0.380 **	0.054	−0.029	−0.079	−0.015	−0.190	−0.083	−0.019
VFA	−0.245 *	−0.109	−0.313 **	−0.298 **	0.149	0.004	−0.046	−0.014	−0.200 *	−0.088	0.046
PSM	0.171	0.215 *	0.315 **	0.392 **	−0.016	0.043	0.100	0.033	0.192	0.095	0.044
WC	−0.278 **	−0.032	−0.199 *	−0.111	0.146	−0.013	−0.013	0.022	−0.140	−0.053	0.051
HC	−0.202 *	0.057	−0.184	−0.079	0.230 *	0.078	0.050	−0.005	−0.134	−0.014	0.157
WHR	−0.274 **	−0.113	−0.144	−0.093	0.006	−0.102	−0.055	0.050	−0.084	−0.053	−0.063
VO_2_max	0.119	0.134	0.242 *	0.318 **	−0.006	−0.083	0.013	−0.002	0.112	0.099	−0.066
RGS	0.035	0.126	0.032	0.050	0.105	0.133	0.173	0.085	0.145	0.122	0.162
SFF	0.034	0.003	0.070	0.044	−0.074	0.014	0.131	0.093	0.198 *	0.017	0.105
GS	−0.132	0.141	0.042	0.150	0.147	0.042	0.115	0.059	0.065	0.111	0.107

* *p* < 0.05, ** *p* < 0.01.

**Table 4:** Multiple regression analysis of quality of life.

Dependent Variable		Unstandardized Coefficients		Standardized Coefficients	95% CI		P
		B	Standard Error	β	Lower	Upper	
HT	Intercept	110.498	24.161		62.575	158.422	0.004
	WC	−0.706	0.242	−0.278	−1.185	−0.226	
GH	Intercept	100.442	12.977		74.703	126.181	0.004
	PBF	−1.449	0.401	−0.337	−2.243	−0.654	
PF	Intercept	56.104	7.493		33.438	68.821	<0.001
	PSM	0.690	0.204	0.321	0.286	1.094	
	VO_2_max	0.278	0.125	0.211	0.030	0.526	
RP	Intercept	−16.492	41.884		−99.568	66.584	0.019
	HC	0.948	0.398	0.230	0.160	1.737	
PCS	Intercept	49.247	13.142		23.180	75.314	0.028
	PSM	0.762	0.343	0.215	0.083	1.441	
VT	Intercept	78.018	5.149		67.805	88.232	0.003
	VFA	−0.081	0.039	−0.200	−0.160	−0.003	

Adjusted for sex, age, comorbidity and degree of liver steatosis.

## Data Availability

The data presented in this study are available on request to qualified researchers from the corresponding author.

## Abbreviations

ALP	alkaline phosphatase
ALT	alanine aminotransferase
AST	aspartate aminotransferase
BMI	body mass index
BP	bodily pain
CAP	continuous attenuation parameter
CRF	cardiorespiratory fitness
FBS	fast glucose sugar
GH	general health
GS	grip strength
HC	hip circumference
HDL-C	high-density lipoprotein cholesterol
HT	health transition
LDL-C	low-density lipoprotein cholesterol
LSM	liver stiffness measurement
MCS	mental component summary score
MH	mental health
PBF	percent body fat
PCS	physical component summary score
PF	physical functioning
PSM	percent skeletal muscle mass
RE	role limitation due to emotional problem
RGS	relative grip strength
RP	role limitation due to physical problems
SF	social functioning
SFF	sitting forward flexion
SF-36	36-item Short Form Health Survey Questionnaire
TC	total cholesterol
TG	triglyceride
VFA	visceral fat area
VO_2_max	maximal oxygen uptake
VT	vitality
WC	waist circumference
WHR	waist-to-hip ratio
γ-GT	γ-glutamyl transferase

## References

[1] Fan J.G., Wei L., Zhuang H., Cai W., Feng Chen D., National Workshop on Fatty Liver and Alcoholic Liver Disease, Chinese Society of Hepatology, Chinese Medical Association, Fatty Liver Disease Expert Committee, Chinese Medical Doctor Association (2019). Guidelines of prevention and treatment of nonalcoholic fatty liver disease (2018, China). J. Dig. Dis..

[2] David K., Kowdley K.V., Unalp A., Kanwal F., Brunt E.M., Schwimmer J.B., NASH CRN Research Group (2009). Quality of life in adults with nonalcoholic fatty liver disease: Baseline data from the nonalcoholic steatohepatitis clinical research network. Hepatology.

[3] Flanagan S., Damery S., Combes G. (2017). The effectiveness of integrated care interventions in improving patient quality of life (QoL) for patients with chronic conditions. An overview of the systematic review evidence. Health Qual. Life Outcomes.

[4] Afendy A., Kallman J.B., Stepanova M., Younoszai Z., Aquino R.D., Bianchi G., Marchesini G., Younossi Z.M. (2009). Predictors of health-related quality of life in patients with chronic liver disease. Aliment. Pharmacol. Ther..

[5] Kim H.J., Chu H., Lee S. (2018). Factors influencing on health-related quality of life in South Korean with chronic liver disease. Health Qual. Life Outcomes.

[6] Huang R., Fan J.G., Shi J.P., Mao Y.M., Wang B.Y., Zhao J.M., Lu L.G., Zhong B.H., Zou Z.S., Xu Y.Q. (2021). Health-related quality of life in Chinese population with non-alcoholic fatty liver disease: A national multicenter survey. Health Qual. Life Outcomes.

[7] Golabi P., Otgonsuren M., Cable R., Felix S., Koenig A., Sayiner M., Younossi Z.M. (2016). Non-alcoholic Fatty Liver Disease (NAFLD) is associated with impairment of Health Related Quality of Life (HRQOL). Health Qual. Life Outcomes.

[8] Ozawa N., Sato K., Sugimura A., Maki S., Tanaka T., Yamamoto K., Ito T., Ishizu Y., Kuzuya T., Honda T. (2021). Quality of Life in patients with nonalcoholic fatty liver disease: Structure and related factors focusing on illness uncertainty. Jpn. J. Nurs. Sci..

[9] Ruiz J.R., Castro-Pinero J., Artero E.G., Ortega F.B., Sjostrom M., Suni J., Castillo M.J. (2009). Predictive validity of health-related fitness in youth: A systematic review. Br. J. Sports Med..

[10] Appelqvist-Schmidlechner K., Vaara J.P., Vasankari T., Hakkinen A., Mantysaari M., Kyrolainen H. (2020). Muscular and cardiorespiratory fitness are associated with health-related quality of life among young adult men. BMC Public Health.

[11] Hao L., Wang Z., Wang Y., Wang J., Zeng Z. (2020). Association between Cardiorespiratory Fitness, Relative Grip Strength with Non-Alcoholic Fatty Liver Disease. Med. Sci. Monit..

[12] American College of Sports Medicine (2017). ACSM’s Exercise Testing and Prescription.

[13] Li L., Wang H.M., Shen Y. (2003). Chinese SF-36 Health Survey: Translation, cultural adaptation, validation, and normalisation. J. Epidemiol. Community Health.

[14] Martinez-Urbistondo D., San Cristobal R., Villares P., Martinez-Gonzalez M.A., Babio N., Corella D., Del Val J.L., Ordovas J.M., Alonso-Gomez A.M., Warnberg J. (2022). Role of NAFLD on the Health Related QoL Response to Lifestyle in Patients With Metabolic Syndrome: The PREDIMED Plus Cohort. Front. Endocrinol. Lausanne.

[15] Alrasheed M., Guo J.J., Lin A.C., Wigle P.R., Hardee A., Hincapie A.L. (2022). The effect of polypharmacy on quality of life in adult patients with nonalcoholic fatty liver disease in the United States. Qual. Life Res..

[16] Kennedy-Martin T., Bae J.P., Paczkowski R., Freeman E. (2017). Health-related quality of life burden of nonalcoholic steatohepatitis: A robust pragmatic literature review. J. Patient Rep. Outcomes.

[17] Ware J.E., Sherbourne C.D. (1992). The MOS 36-item short-form health survey (SF-36): I. Conceptual framework and item selection. Med. Care.

[18] General Administration of Sport of China (2003). Handbook of National Physical Fitness Standards.

[19] Cho J., Lee I., Park D.H., Kwak H.B., Min K. (2021). Relationships between Socioeconomic Status, Handgrip Strength, and Non-Alcoholic Fatty Liver Disease in Middle-Aged Adults. Int. J. Environ. Res. Public Health.

[20] Eslam M., Sarin S.K., Wong V.W., Fan J.G., Kawaguchi T., Ahn S.H., Zheng M.H., Shiha G., Yilmaz Y., Gani R. (2020). The Asian Pacific Association for the Study of the Liver clinical practice guidelines for the diagnosis and management of metabolic associated fatty liver disease. Hepatol. Int..

[21] Ofusa Y., Golding L.A. (2000). YMCA Fitness Testing and Assessment Manual.

[22] Kaminsky L.A., Arena R., Myers J. (2015). Reference Standards for Cardiorespiratory Fitness Measured with Cardiopulmonary Exercise Testing: Data From the Fitness Registry and the Importance of Exercise National Database. Mayo Clin. Proc..

[23] Xue R., Li Q., Geng Y., Wang H., Wang F., Zhang S. (2021). Abdominal obesity and risk of CVD: A dose-response meta-analysis of thirty-one prospective studies. Br. J. Nutr..

[24] Sharma M., Kulkarni A., Kumar P., Nori V.B., Jagtap N., Gupta R., Reddy D.N., Rao P.N. (2020). Difference in lifestyle and metabolic profile of non-alcoholic fatty liver disease with raised alanine amino-transferases between obese and non-overweight subjects. Sci. Rep..

[25] Alberti K.G., Zimmet P., Shaw J., Group I.D.F.E.T.F.C. (2005). The metabolic syndrome--a new worldwide definition. Lancet.

[26] Alberti K.G., Zimmet P.Z. (1998). Definition, diagnosis and classification of diabetes mellitus and its complications. Part 1: Diagnosis and classification of diabetes mellitus provisional report of a WHO consultation. Diabet. Med..

[27] Joint Committee for Guideline Revision (2018). 2016 Chinese guidelines for the management of dyslipidemia in adults. J. Geriatr. Cardiol..

[28] Fang J., Alderman M.H. (2000). Serum uric acid and cardiovascular mortality the NHANES I epidemiologic follow-up study, 1971–1992. National Health and Nutrition Examination Survey. JAMA.

[29] James P.A., Oparil S., Carter B.L., Cushman W.C., Dennison-Himmelfarb C., Handler J., Lackland D.T., LeFevre M.L., MacKenzie T.D., Ogedegbe O. (2014). 2014 evidence-based guideline for the management of high blood pressure in adults: Report from the panel members appointed to the Eighth Joint National Committee (JNC 8). JAMA.

[30] Ross R., Blair S.N., Arena R., Church T.S., Despres J.P., Franklin B.A., Haskell W.L., Kaminsky L.A., Levine B.D., Lavie C.J. (2016). Importance of Assessing Cardiorespiratory Fitness in Clinical Practice: A Case for Fitness as a Clinical Vital Sign: A Scientific Statement From the American Heart Association. Circulation.

[31] Krasnoff J.B., Painter P.L., Wallace J.P., Bass N.M., Merriman R.B. (2008). Health-related fitness and physical activity in patients with nonalcoholic fatty liver disease. Hepatology.

[32] Afolabi P.R., Scorletti E., Calder P.C., Byrne C.D. (2020). Factors independently associated with cardiorespiratory fitness in patients with non-alcoholic fatty liver disease. Liver Int..

[33] Shin H.S. (2020). Association between periodontal status and non-alcoholic fatty liver disease in a Korean adult population: A nationwide cross-sectional study. J. Periodontol..

[34] Pratesi A., Tarantini F., Di Bari M. (2013). Skeletal muscle: An endocrine organ. Clin. Cases Miner. Bone Metab..

[35] Kim G., Lee S.E., Lee Y.B., Jun J.E., Ahn J., Bae J.C., Jin S.M., Hur K.Y., Jee J.H., Lee M.K. (2018). Relationship Between Relative Skeletal Muscle Mass and Nonalcoholic Fatty Liver Disease: A 7-Year Longitudinal Study. Hepatology.

[36] Petermann-Rocha F., Gray S.R., Forrest E., Welsh P., Sattar N., Celis-Morales C., Ho F.K., Pell J.P. (2022). Associations of muscle mass and grip strength with severe NAFLD: A prospective study of 333,295 UK Biobank participants. J. Hepatol..

[37] Battista F., Ermolao A., van Baak M.A., Beaulieu K., Blundell J.E., Busetto L., Carraca E.V., Encantado J., Dicker D., Farpour-Lambert N. (2021). Effect of exercise on cardiometabolic health of adults with overweight or obesity: Focus on blood pressure, insulin resistance, and intrahepatic fat-A systematic review and meta-analysis. Obes. Rev..

[38] Nuzzo J.L. (2020). The Case for Retiring Flexibility as a Major Component of Physical Fitness. Sports Med..

[39] The World Health Organization (1998). Quality of Life Assessment (WHOQOL): Development and general psychometric properties. Soc. Sci. Med..

[40] Samala N., Desai A., Vilar-Gomez E., Smith E.R., Gawrieh S., Kettler C.D., Pike F., Chalasani N. (2020). Decreased Quality of Life Is Significantly Associated With Body Composition in Patients With Nonalcoholic Fatty Liver Disease. Clin. Gastroenterol. Hepatol..

[41] Younossi Z.M., Stepanova M., Lawitz E.J., Reddy K.R., Wai-Sun Wong V., Mangia A., Muir A.J., Jacobson I., Djedjos C.S., Gaggar A. (2019). Patients With Nonalcoholic Steatohepatitis Experience Severe Impairment of Health-Related Quality of Life. Am. J. Gastroenterol..

[42] Gerber L.H., Weinstein A.A., Mehta R., Younossi Z.M. (2019). Importance of fatigue and its measurement in chronic liver disease. World J. Gastroenterol..

